# A distributed framework for aligning short reads to genomes

**DOI:** 10.1186/1471-2105-15-S10-P22

**Published:** 2014-09-29

**Authors:** Shanshan Guo, Vinthuy Phan

**Affiliations:** 1 Department of Computer Science, University of Memphis, Memphis, TN 38152, USA

## Background

Computational methods that employ next-generation sequencing technologies often depend on the alignment of short reads [1] to genomes. In a typical workflow, such methods might require millions of independent alignment operations. Although using a high-performance cluster (HPC) to distribute these computational independent tasks can speed up the process significantly, a HPC can be expensive, wasteful and sometime not a feasible solution. We propose a distributed framework that aims specifically at distributing the task of aligning short reads to genomes to multiple machines efficiently and effectively. This framework aims to be simple to set up and grow.

## Materials and methods

To accomplish this, we introduce the framework using the Go programming language, which has primitive support for concurrent computation, and utilizes a high performance network library called ZeroMQ [2,3] for effective distribution of queries. Specifically, we use the Pipeline pattern from ZeroMQ. This pattern includes three main parts: (1) ventilator (which distributes reads to workers), (2) worker (which does the main computation and sends results to a sink) and (3) sink (which collects results from workers). There are three stages in our design. In the listening stage, the system sets up. The ventilator sends the REQ message including other important information to workers. The workers load the index into the RAM. In the query stage, the ventilator distributes the reads to the workers. The workers work on aligning the reads to the index loaded in the listening stage. In the last stage, the system closes. The ventilator sends an END message to the workers after it distributes all the reads so that workers can close sockets after processing all reads.

## Conclusions

Simulation showed that the running time of alignment decreased linearly with the number of the workers. This system is easy to use and deploy.

## References

[B1] Morozova O Marra MA Applications of next-generation sequencing technologies in functional genomics Genomics 2008 92 5 255 264 18703132 10.1016/j.ygeno.2008.07.001

[B2] Hintjens P Messaging for many applications 2013 ZeroMQ: Sebastpol: O'Reilly Media, Inc

[B3] An Intro to ZeroMQ(ØMQ) On Ubuntu 12.04 http://babounehacks.blogspot.com/

